# Changes in physicochemical and microbiological properties of isoflavone-treated dry-cured sausage from sulfur-fed pork during storage

**DOI:** 10.1186/2055-0391-56-21

**Published:** 2014-10-11

**Authors:** Ji-Han Kim, Chang-Won Pyun, Go-Eun Hong, Soo-Ki Kim, Cheul-Young Yang, Chi-Ho Lee

**Affiliations:** Konkuk University of Food Science & Technology, Seoul, 143-701 Republic of Korea; Konkuk University of Animal Science & Technology, Seoul, 143-791 Republic of Korea; Eulji University of Food Technology & Services, Sung-nam, 461-713 Republic of Korea

**Keywords:** Isoflavone, Dry-cured sausage, Sulfur, Lipid oxidation, Storage

## Abstract

This study was performed to investigate the physicochemical and microbiological properties of isoflavone-treated dry cured sausage from sulfur fed pork (0.3%) during storage at 15°C for 45 days. Groups were divided into three treatments: dry-cured sausages produced with pork fed general diet as the control group (CON), sulfur-fed pork (SUL) and isoflavone-(0.25%) treated sulfur-fed pork (ISO). Moisture content in all groups decreased dramatically from 55–57% to 10–11% during storage, whereas crude protein, crude fat, and ash content increased (*P* < 0.05). The ISO group showed excellent antioxidant effect compared to CON during storage. Redness and lightness of ISO was higher than that of CON during storage. VBN in the ISO group was significantly lower than that in the CON and SUL treatments during 30 and 45 days of storage (*P* < 0.05). A total plate count of ISO was significantly lower than that of CON at 45 days (*P* < 0.05). In this study, adding isoflavone to meat products indicated prevention of lipid oxidation and improved color stability in meat products.

## Background

Functional foods have beneficial effects besides merely supplying nutrition. Addition of functional materials can extend shelf-life and prevent rancidity of products as well as modulate various body functions [1]. Traditional fermented foods have been shown to possess many of these beneficial effects, and their extracts are often applied to other foods after extraction and purification [2].

Dry-cured pork sausage is made by thorough mixing with lean pork back fat and other non-meat ingredients, including salt, nitrite, spices, and a starter. Each additive is useful for preserving flavor, color, and water-holding capacity in meat products. On average, dry-cured pork sausage is 70–80% lean and 20–30% lard, which includes 43% saturated fatty acids (SFAs), 47% monounsaturated fatty acids, and 10% polyunsaturated fatty acids. Half of the fatty acids in pork fat are SFAs, which significantly contribute to cardiovascular disease [3].

Lipid oxidation, which has a negative influence on meat product quality [4], can be inhibited by natural or artificial antioxidants [5]. Isoflavones, which are abundantly available in soybeans, are natural antioxidative materials. Isoflavones reduce blood cholesterol and LDL-cholesterol oxidation through antioxidant and free radical scavenging activities [6]. Further, lipid oxidation must be considered during the drying period of dry-cured sausage due to its high lipid content. Therefore, many researchers have investigated ways to prevent lipid oxidation in order to increase the quality and safety of these meat products.

The aim of this study was to investigate the effects of isoflavones on the physicochemical and microbiological properties of dry-cured sausage during the drying period.

## Methods

### Formula and chemical composition of diet

A total of 90 three-way crossbred pigs (Landrace, Duroc, and Yorkshire) from Yang-ju Federation of Livestock Cooperatives in the Republic of Korea were used. Experimental protocol was approved by the animal care committee of Konkuk University of Seoul, Republic of Korea. Detailed feeding and rearing procedures as well as the composition of the feed were reported previously [7]. The pigs, which weighed 110 kg each at shipment, were divided into three groups based on the level of dietary processed sulfur (0%, 0.3%) fed for 3 months before shipment. Processed sulfur was used as received from Ebatha Co., Ltd. The control (CON) was not supplied processed sulfur, whereas the SUL group was supplied with processed sulfur at 3 g/kg feed for 3 months before shipment.

### Weight, average daily feed intake, weight gain, feed efficiency, and carcass grade

#### Dry-cured sausage preparation

Pork meat and fat were trimmed off with a sterile knife and refrigerated overnight at about 4°C. The composition of the meat mixture is shown in Table 1. The basic formula was 75% lean pork and 25% back fat with curing ingredients. The trimmed meat was ground using a 2.7 mm plate meat mincer and mixed with other curing ingredients. Starter culture at 0.25% (*Staphylococcus carnosus* M17: *Pediococcus pentosaceus* ATCC 33314 = 1:1) was added into the mixed sample. This original mixture was then split into batches, after which 0.25% isoflavone powder obtained from SOLGAR® (Seoul, Republic of Korea) was added to the ISO group. Finally, the minced meat was filled into collagen casings (150 mm long, 30 mm diameter), and all samples were ripened for 45 days at 15 ± 2°C and a relative humidity of 80 ± 3% in a chamber. Sampling was performed by randomly choosing each sausage group after 0, 15, 30, and 45 days for physicochemical and microbiological analyses [8].

**Table 1:** **Common components of the meat mixture**

	Ingredients	Percentage (%)		
		CON	SUL	ISO
Materials	Lean pork	75	75	75
	Pork back fat	25	25	25
	Salt	2.8	2.8	2.8
	Black pepper	0.25	0.25	0.25
Pickle	Rosemary	0.05	0.05	0.05
	Sodium nitrite	0.02	0.02	0.02
	Glucose	1	1	1
	Isoflavone			0.25
Starter culture	*Staphylococcus carnosus* M17	0.25	0.25	0.25
	*Pediococcus pentosaceus* ATCC 33314			

CON and SUL groups were divided from the level of processed sulfur. Sulfur levels in diets were controlled by crude fiber content. (SUL: 0.3% sulfur).

#### Physicochemical analysis of dry-cured sausage

Proximate compositions (moisture, crude fat, crude protein, and ash) of the sausage samples were determined by the AOAC method [9]. Water activities (a_w_) of the samples were determined using a water activity measuring device (Aquaspector, AQS-31, NAGY, Gaeurfelden, Germany) The a_w_ values were determined in triplicate in order to optimize the weights of samples at 25°C until equilibrium was reached.

The pH was measured using a pH meter (pH 900, Precisa Co, Deitikon, Switzerland) in a slurry made by homogenizing 2 g of sample with 18 mL of distilled water for 90 sec using a Bag mixer 400 (Interscience Co, St Nom la Bretêche, France).

Thiobarbituric acid (TBA) values of dry-cured sausage stored for different times were determined by following a modified method of Witte et al. [10]. The absorbance of the supernatant was measured at 532 nm using a spectrophotometer (Optizen 2120UV, Mecasys, Seoul, Korea). Results are expressed as mg malonaldehyde (MDA)/kg sample. Volatile basic nitrogen (VBN) was determined by the micro-diffusion method of Conway [11] for dry-cured sausage stored for different times. Results were expressed as VBN value % mg (mg/100 g meat). The color values of dry-cured sausages were expressed as Hunter L-, a-, and b-values using a Handy colorimeter (NR-300, Nippon Denshoku, Tokyo, Japan). All experiments were performed in triplicate.

#### Microbiological analysis

Sample (2 g) and 0.85% NaCl (18 mL) in sterile deionized water were transferred to a sterile stomacher bag and homogenized for 90 sec using a bag mixer (Interscience Co, France). A 10^-1^ dilution was then used for subsequent serial dilutions. An aliquot (0.1 mL) of the appropriate dilution of sample was spread in triplicate onto agar plates. After serial dilution, the solution was inoculated onto Petrifirm Aerobic Count Plates (3 M, Korea) and cultured for 48 h at 35°C, after which the colony number was converted into a log value. The samples were treated as described above and inoculated onto Petrifirm *E. coli* O157:H7 Count Plates (3 M, Korea) for 48 h at 35°C. Lactic acid bacteria were treated as described above, inoculated onto MRS agar (OXOID, England), and then cultured for 24 h at 35°C. *Staphylococcus aureus* bacteria were treated as described above, inoculated onto Baird-Parker agar (OXOID, England), and then cultured for 24 h at 35°C. *Salmonella* bacteria were treated as described above, inoculated onto MacConkey agar (Difco, USA), and then cultured for 24 h at 35°C.

#### Statistical analysis

Analysis of variance (ANOVA) was conducted to determine significant differences among the groups and storage times. All analyses were performed on all variables using the General Linear Model (GLM) procedure of SAS version 9.2 (SAS Institute Inc., Cary, NC, USA). All analyses were conducted in triplicate, and significant differences were detected using Duncan’s multiple range test (p < 0.05).

## Result and discussion

### Physicochemical qualities

The initial moisture content of all dry-cured ham samples was 54.71–57.97%, which declined to 10.4–11.56% in the final product (Table 2). In the initial phase, moisture contents of the ISO and SUL groups were significantly higher than that of the CON (p < 0.05). This result could be due to the higher water-holding capacity of sulfur-fed pigs [12]. The reduction of moisture content was accompanied by elevation of the other components during the drying period. Crude fat contents of the SUL and ISO groups were lower than that of the CON during storage. Promotion of lipid metabolism and reduction of intramuscular fat have been observed in animals fed dietary sulfur [13]. Crude protein contents of the CON, SUL, and ISO groups increased significantly (p < 0.05) until day 30, followed by a decrease. The processing time of dry-cured ham depends on the product. Specifically, longer processing time results in more intense muscle proteases activities, leading to extensive protein breakdown [14].

**Table 2:** **Effect of isoflavone on the change in moisture, crude protein, crude fat, and ash contents in dry-cured pork sausage during storage (%)**

Proximate composition (%)	Storage period (days)	Treatments			SEM	P-value	
		CON	SUL	ISO		Treatment	Storage
Crude protein	0	17.2^NS B^	16.9^B^	17.4^B^	0.45	0.76	***
	15	31.0^NS A^	31.6^A^	31.9^A^	2.63	0.95	
	30	38.3^NS C^	38.5^A^	37.6^C^	2.37	0.77	
	45	36.8^a C^	34.2^ab A^	32.6^b A^	1.48	*	
Crude fat	0	19.8^a C^	16.3^b^ ^C^	17.9^ab C^	0.93	0.1	***
	15	38.4^NS AB^	37.2^B^	38.6^B^	0.46	0.08	
	30	39.9^NS A^	38.4^B^	38.3^B^	1.43	0.18	
	45	45.3^NS^ ^A^	43.3^A^	44.8^A^	1.94	0.44	
Moisture	0	55.7^b A^	57.3^a A^	57.97^a^ ^A^	0.47	*	***
	15	18.2^NS B^	18.1^B^	18.03^B^	0.76	0.95	
	30	11.2^NS C^	12.1^C^	12.40^C^	2.94	0.73	
	45	11.5^NS C^	11.1^C^	10.44^C^	0.51	0.24	
Ash	0	3.15^b B^	3.64^a C^	3.27^b^ ^C^	0.03	***	***
	15	6.32^NS A^	6.23^B^	6.46^B^	0.29	0.86	
	30	6.15^b A^	7.22^a A^	5.92^c B^	0.33	0.07	
	45	6.65^NS A^	7.52^A^	7.35^A^	0.66	0.44	

1) CON, Dry-cured sausage processed with 100% commercial diet fed pork; SUL, Dry-cured sausage processed with 0.3% sulfur fed pork; ISO, 0.25% isoflavone + dry-cured sausage processed with 0.3% sulfur fed pork. 2)^NS^; Not significant; a-c means within a row with different letters are significantly different, P < 0.05; A-D means within a column with different letters are significantly different, P < 0.05. Significance level of treatment is given by ***(P <0.001); **(P<0.01); *(P<0.05)*All values are mean ± standard deviation (n = 3).

### pH value and water activity (a_w_)

The pH values of all groups decreased during 15 days of storage but increased during the experimental period (Table 3). The pH value of the ISO group was significantly higher than that of the CON group at the beginning of the drying process (p < 0.05), possibly due to inhibition of lactic acid production caused by addition of isoflavones [15]. Reduction of the pH values of all samples during drying can be mainly attributed to lactic acid bacterial growth in the initial phase [16]. Many researchers have indicated that production of lactic acid in tissue leads to pH reduction in meat, resulting in less water [17]. After 15 days, elevation of the pH value could also be due to the accumulation of non-protein nitrogen or amino acid catabolic products [18].

**Table 3:** **Effect of isoflavone on the pH and water activity (a**_**w**_ **) change in dry-cured pork sausage during the ripening period**

	Storage period (days)	Treatments			SEM	P-value	
		CON	SUL	ISO		Treatment	Storage
pH	0	6.00^b A^	6.03^ab A^	6.06^a A^	0.012	*	***
	15	5.91^NS B^	5.92^B^	5.99^B^	0.008	0.064	
	30	6.17^NS C^	6.16^C^	6.10^C^	0.038	0.369	
	45	6.23^b D^	6.26^a D^	6.23^b D^	0.027	0.23	
a_w_	0	0.97^NS A^	0.97^A^	0.99^A^	0.001	0.123	***
	15	0.69^b B^	0.72^a B^	0.72^a B^	0.009	0.096	
	30	0.69^b B^	0.71^a B^	0.71^a B^	0.005	*	
	45	0.65^c C^	0.68^a C^	0.66^b C^	0.002	***	

1) CON, Dry-cured sausage processed with 100% commercial diet fed pork; SUL, Dry-cured sausage processed with 0.3% sulfur fed pork; ISO, 0.25% isoflavone + dry-cured sausage processed with 0.3% sulfur fed pork. 2)^NS^; Not significant; a-c means within a row with different letters are significantly different, P < 0.05; A-D means within a column with different letters are significantly different, P < 0.05. Significance level of treatment is given by ***(P <0.001); **(P<0.01); *(P<0.05) *All values are mean ± standard deviation (n = 3).

The a_w_ of all three groups decreased significantly during 45 days of storage (p < 0.05, Table 3). The a_w_ of the SUL and ISO groups were significantly higher than that of the CON during storage (p < 0.05). Meat products can be classified as intermediate moisture meats when their a_w_ values range from 0.60 to 0.90 [19].

### Thiobarbituric acid reactive substances (TBARS)

TBA value of dry-cured pork sausage is shown in Table 4. TBA values of all samples increased significantly during storage (p < 0.05), which corroborates the results of a previous study [20]. The ISO and SUL groups showed significantly lower TBA values than that of the CON (p < 0.05). This result could be attributed to the antioxidant effects of sulfur-containing compounds and isoflavones [12, 21].

**Table 4:** **Effect of isoflavone on the change in thiobarbituric acid values in dry-cured pork sausage during the ripening period (Malondialdehyde mg/kg)**

Storage (days)	Treatment			SEM	P-value	
	CON	SUL	ISO		Treatment	Storage
0	0.14^a C^	0.09^b C^	0.11^ab C^	0.01	*	***
15	0.18^a B^	0.16^b B^	0.15^b B^	0.007	*	
30	0.23^a A^	0.21^ab A^	0.20^b A^	0.031	0.19	
45	0.20^a A^	0.16^b B^	0.11^c C^	0.022	0.19	

1) CON, Dry-cured sausage processed with 100% commercial diet fed pork; SUL, Dry-cured sausage processed with 0.3% sulfur fed pork; ISO, 0.25% isoflavone + dry-cured sausage processed with 0.3% sulfur fed pork. 2)^NS^; Not significant; a-c means within a row with different letters are significantly different, P < 0.05; A-D means within a column with different letters are significantly different, P < 0.05. Significance level of treatment is given by ***(P <0.001); **(P<0.01); *(P<0.05)* All values are mean ± standard deviation (n = 3).

Song et al [22] reported that dietary sulfur administered to pigs causes an increase in sulfur-containing antioxidants. Addition of isoflavones reduced the TBA value compared to that of the CON and SUL groups. According to Jiang et al. [23], this result could be due to addition of isoflavones, which increases pH and prevents lipid oxidation. Maximum MDA content values were observed in all samples on day 30, after which values decreased. This result could be attributed to decomposition of MDA by bacteria, which can selectively breakdown and utilize carbonyl compounds such as MDA [24, 25]. The reduced TBA value by day 45 could be due to lipid oxidation products such as organic acids and alcohols, which did not form colored compounds when TBA reacted with MDA [26].

### Volatile basic nitrogen (VBN)

The VBN values of all dry-cured sausages increased significantly (p < 0.05) during storage (Table 5). The range of VBN values is 7–18 mg % in Chinese style dry-cured sausage during the ripening period [27]. The VBN value represents the total growth of aerobic bacteria in meat products [28]. In our results, the VBN value of the ISO sample was significantly lower than that of the CON during days 30–45. This result can be attributed to addition of isoflavones, which have antimicrobial effects [28]. The VBN value may also be an indicator of spoilage [21, 29]. VBN values decreased in all samples on the last day of the ripening process compared to day 30, suggesting inhibition of VBN by lactic acid bacteria [30].

**Table 5:** **Effect of isoflavone on the change in volatile basic nitrogen (VBN) in dry-cured pork sausage during the ripening period (mg %)**

Storage (days)	Treatment			SEM	P-value	
	CON	SUL	ISO		Treatment	Storage
0	8.2^NS C^	8.9^B^	8.2^B^	0.304	0.22	***
15	10.9^NS B^	11.0^A^	10.8^A^	0.459	0.93	
30	11.8^a A^	11.4^a A^	10.1^b A^	0.296	*	
45	9.0^a C^	8.6^ab B^	7.8^b B^	0.216	*	

1) CON, Dry-cured sausage processed with 100% commercial diet fed pork; SUL, Dry-cured sausage processed with 0.3% sulfur fed pork; ISO, 0.25% isoflavone + dry-cured sausage processed with 0.3% sulfur fed pork. 2)^NS^; Not significant; a-c means within a row with different letters are significantly different, P < 0.05; A-D means within a column with different letters are significantly different, P < 0.05. Significance level of treatment is given by ***(P <0.001); **(P<0.01); *(P<0.05) *All values are mean ± standard deviation (n = 3).

### Color

Changes in the color of dry-cured pork sausage during storage are shown in Table 6. The rate of meat product discoloration is related to oxidation and reduction of metmyoglobin in meat as well as the effects of its three derivatives (myoglobin, deoxymyoglobin, oxymyoglobin) on L^*^, a^*^, and b^*^ values [31, 32].

**Table 6:** Effect of isoflavone on the change in lightness coordinate (L*), redness coordinate (a*), and yellowness coordinate (b*) in dry-cured pork sausage during the ripening period

L* (Lightness)	Treatment			SEM	P-value	
Storage (days)	CON	SUL	ISO		Treatment	Storage
0	62.1^b A^	64.7^a A^	64.2^a A^	0.447	*	***
15	48.6^NS D^	47.2^C^	49.4^D^	0.633	0.130	
30	49.1^NS^ ^C^	50.4^B^	51.4^C^	0.692	0.134	
45	51.2^b B^	52.8^ab B^	54.4^a B^	0.806	0.073	
a* (redness)	CON	SUL	ISO			
Storage (days)						
0	6.1^NS C^	6.8^D^	6.5 ^C^	0.361	0.435	***
15	12.6^NS A^	11.5^A^	12.0 ^A^	0.567	0.453	
30	10.2^NS A^	10.6^B^	11.0^A^	0.457	0.518	
45	8.1^NS B^	9.1^C^	9.1^B^	0.555	0.412	
b* (yellowness)	CON	SUL	ISO			
Storage (days)						
0	13.2^b C^	16.9^a B^	14.1^b D^	0.470	**	***
15	17.6^a A^	15.4^b A^	18.5^a A^	0.481	**	
30	15.7^b B^	15.4^b A^	17.3^a B^	0.337	*	
45	14.8^b B^	15.3^ab A^	16.8^a C^	0.436	*	

1) CON, Dry-cured sausage processed with 100% commercial diet fed pork; SUL, Dry-cured sausage processed with 0.3% sulfur fed pork; ISO, 0.25% isoflavone + dry-cured sausage processed with 0.3% sulfur fed pork. 2)^NS^; Not significant; a-c means within a row with different letters are significantly different, P < 0.05; A-D means within a column with different letters are significantly different, P < 0.05. Significance level of treatment is given by ***(P <0.001); **(P<0.01); *(P<0.05) *All values are mean ± standard deviation (n = 3).

The lightness (L*) values of all samples were between 62–64 and decreased (p < 0.05) to about 15 during the ripening period. This reduction of the L-value resulted in dark coloration due to browning reaction in all dry-cured hams. It was previously reported that metmyoglobin generated by pigment oxidation reduces lightness value while pigment oxidation shows a positive relationship with lipid oxidation during the ripening period [33, 34]. Therefore, the high lightness value of the ISO group could be due to prevention of pigment oxidation during storage. The redness (a^*^) values of all samples increased significantly compared to that of the initial meat product (p < 0.05), but a* values decreased slightly in all samples after 15 days. Similarly, Kayaardı and Gök [33] reported that a-values of dry-cured ham increase during the initial ripening period, after which they decrease during further ripening. Additionally, pH and redness have a proportional relationship in meat products. A similar result was observed by Fu et al [35]. However, no significant differences were observed among the three groups. Redness (a*) was not affected by isoflavone or sulfur content.

The yellowness (b*) value of the meat was between 13–18 during ripening. All b* values decreased slightly during the ripening period. This result corroborates the yellowness of Spanish sausage, which decreases during the ripening period [36]. No significant differences were observed among the groups during fermentation.

### Microbiological assessment

Microbiological analyses of the dry-cured sausages during the 45-day storage period are shown in Table 7. Total plate counts increased during the initial phase and then decreased until day 45. Total aerobic bacterial counts were significantly lower in dry-cured sausage containing isoflavones compared to other sausages during the entire ripening process. This result may be due to the antimicrobial activity of isoflavonoids, as many researchers have reported that isoflavones are representative flavonoids with antibacterial activity [37, 38]. The number of lactic acid bacteria increased by day 15 and then tended to decrease in all dry-cured sausages until day 45. Visible lactic acid bacteria counts in the ISO group were significantly higher than those in the other samples by day 30. Other studies have also found that conversion of isoflavone glycosides to aglycones has beneficial effects on the growth of *Lactobacillus* [39]. The results of the microbial analysis during sausage fermentation can be a measure of the quality of meat products such as pH and physicochemical properties [40].

**Table 7:** **Effect of isoflavone on the change in microbial counts in dry-cured pork sausage during the ripening period (log CFU/g)**

Composition	Storage (days)	Treatment			SEM	P-value	
		CON	SUL	ISO		Treatment	Storage
Total aerobes	0	5.8^NS B^	5.8^B^	5.8^B^	0.044	**	***
	15	7.3^a A^	7.2^ab A^	7.1^b A^	0.027	*	
	30	4.6^NS C^	4.7^C^	4.3^C^	0.179	0.33	
	45	4.7^a C^	4.7^a C^	4.3^b C^	0.179	0.97	
Lactic acid bacteria	0	5.6^NS B^	5.3^B^	5.4^B^	0.018	**	***
	15	5.8^b A^	5.9^b A^	6.0^a A^	0.104	**	
	30	4.7^NS C^	5.1^C^	4.6^C^	0.184	0.13	
	45	4.5^NS C^	4.5^C^	4.5^C^	0.097	0.75	
*E. coli* 0157:H7	0	-	-	-			
	15	-	-	-			
	30	-	-	-			
	45	-	-	-			
*Staphylococcus aureus*	0	-	-	-			
	15	-	-	-			
	30	-	-	-			
	45	-	-	-			
*Salmonella* spp.	0	-	-	-			
	15	-	-	-			
	30	-	-	-			
	45	-	-	-			

1) CON, Dry-cured sausage processed with 100% commercial diet fed pork; SUL, Dry-cured sausage processed with 0.3% sulfur fed pork; ISO, 0.25% isoflavone + dry-cured sausage processed with 0.3% sulfur fed pork. 2)^NS^; Not significant; a-c means within a row with different letters are significantly different, P < 0.05; A-D means within a column with different letters are significantly different, P < 0.05. *All values are mean ± standard deviation (n = 3).

^a,b^ means with different superscripts in the same row are significantly different (*p* < 0.05).

Significance level of treatment is given by ***(P <0.001); **(P<0.01); *(P<0.05). *All values are mean ± standard deviation (n = 3).

## Conclusion

The results demonstrated the antioxidative and antimicrobial effects of isoflavones on dry-cured sausage during ripening period (15°C). The combination of isoflavones and sulfur-fed pork showed the best results in terms of a_w_ and TBA values, which are the main concerns in dry-cured meat products during storage. These beneficial effects are related to inhibition of lipid oxidation and product spoilage. Isoflavones may have commercial potential for extending the shelf-life of meat products without other additives such as nitrites. Further research could demonstrate the antimicrobial effect of isoflavones on lactic acid bacteria during meat fermentation by optimizing the quantities of isoflavones used in this study.
